# The Impact of Stress and Social Determinants on Diet in Cardiovascular Prevention in Young Women

**DOI:** 10.3390/nu16071044

**Published:** 2024-04-03

**Authors:** Francesca Coppi, Valentina Bucciarelli, Kateryna Solodka, Valentina Selleri, Giada Zanini, Marcello Pinti, Milena Nasi, Beatrice Salvioli, Savina Nodari, Sabina Gallina, Anna Vittoria Mattioli

**Affiliations:** 1 Department of Medical and Surgical Sciences for Children and Adults, University of Modena and Reggio Emilia, 41121 Modena, Italy; francesca.coppi@unimore.it; 2 Cardiovascular Sciences Department, Azienda Ospedaliero-Universitaria delle Marche, 60166 Ancona, Italy; valentina.bucciarelli@ospedaliriuniti.marche.it; 3 Istituto Nazionale per le Ricerche Cardiovascolari, 40126 Bologna, Italymarcello.pinti@unimore.it (M.P.); sabina.gallina@unich.it (S.G.); 4 Department of Life Sciences, University of Modena and Reggio Emilia, 41121 Modena, Italy; valentina.selleri@unimore.it (V.S.); giada.zanini@unimore.it (G.Z.); 5 Department of Surgical, Medical and Dental Sciences, University of Modena and Reggio Emilia, 41121 Modena, Italy; milena.nasi@unimore.it; 6 Department of Quality of Life Sciences, University of Bologna, 40126 Bologna, Italy; beatrice.salvioli@unibo.it; 7 Department of Medical and Surgical Specialties, Radiological Sciences and Public Health, University of Brescia, 25123 Brescia, Italy; savina.nodari@unibs.it; 8 Department of Neuroscience, Imaging and Clinical Sciences, “G. d’Annunzio” University of Chieti-Pescara, 66013 Chieti, Italy

**Keywords:** women, cardiovascular risk, pregnancy, Life’s Essential 8, monitoring, physical activity, diet

## Abstract

The prevention of cardiovascular diseases is a fundamental pillar for reducing morbidity and mortality caused by non-communicable diseases. Social determinants, such as socioeconomic status, education, neighborhood, physical environment, employment, social support networks, and access to health care, play a crucial role in influencing health outcomes and health inequities within populations. Social determinants and stress in women are interconnected factors that can significantly impact women’s health and well-being. Pregnancy is a good time to engage young women and introduce them to beneficial behaviors, such as adopting essential life skills, especially diet, and learning stress management techniques. Stress influences diet, and women are more likely to engage in unhealthy eating behaviors such as emotional eating or coping with stress with food. Strong action is needed to improve women’s lifestyle starting at a young age considering that this lays the foundation for a lower cardiovascular risk in adults and the elderly. The objective of this review is to examine cardiovascular primary prevention in young healthy women, focusing particularly on unresolved issues and the influence of social determinants, as well as the correlation with stressors and their influence on diet.

## 1. Introduction

Cardiovascular disease (CVD) in women presents with clinical pictures different from those in men; exposure to traditional risk factors is enriched with the action of risk factors specific to the female sex, and the response to drugs also seems to be influenced by the genetic differences. All this involves a different approach both in primary prevention and in the diagnosis and treatment of cardiovascular disease in women [1,2]. New evaluation algorithms have been introduced; however, since cardiovascular risk develops significantly after menopause, it is widely believed that women do not develop cardiovascular events before menopause [2]. This is not true because even women before menopause can develop cardiovascular (CV) events. This determines that prevention must start early as atherosclerosis is a slow and progressive phenomenon over time starting in the early stages of life [1,2,3,4,5].

Prevention of CVD presents both challenges and opportunities for disease prevention due to its multifaceted nature and significant impact on public health. The development of cardiovascular diseases is influenced by a multitude of risk factors and therefore requires global and multifaceted approaches. Subjects differ in their genetic predispositions, lifestyles, and environmental exposures, leading to variability in CVD risk profiles and responses to preventive interventions. Adapting prevention strategies to individual needs and characteristics can be difficult. The first obstacle is determined by the long period of development and latency of CVD, during which individuals can remain asymptomatic despite the progression of the underlying disease. This makes early diagnosis and intervention difficult [2,5].

Furthermore, socioeconomic factors such as income, education, access to healthcare, and living conditions significantly influence the risk and outcomes of cardiovascular disease. Addressing socioeconomic disparities in cardiovascular disease prevention requires addressing broader social determinants of health (SDOH) [1,2].

The objective of this review is to examine cardiovascular primary prevention in young healthy women, focusing particularly on unresolved issues and the influence of social determinants, as well as the correlation with stressors and their influence on diet. This review focuses on primary prevention in young women without any known heart disease and on the cardiovascular health of young, healthy women.

## 2. Gender Differences in CVD Prevention

### 2.1. The Long Journey of Atherosclerosis: Gender Differences

Although the clinical symptoms caused by atherosclerosis appear in middle and late adulthood, it is known that there is a long phase of development of the asymptomatic pathology, which begins in the first years of life, often during childhood. In most children, atherosclerotic vascular changes are mild and can be minimized or prevented with a healthy lifestyle [5,6,7,8].

Thus, events occurring in the early phases of life can profoundly impact future ASCVD (atherosclerotic cardiovascular disease) risk. For these reasons, ASCVD prevention should commence as early as possible to mitigate the onset of predisposing factors that could affect individual health later in life. Early detection and management of risk factors are essential in preventing or slowing the progression of atherosclerosis in young women and reducing the risk of cardiovascular events in adulthood and old age [4,5,6,7,8]. The initial stage of atherosclerosis is identified as a “fatty streak”, commonly found in children and young individuals. These streaks comprise monocyte-derived macrophages and T lymphocytes, which are inflammatory cells that accumulate within arterial walls and serve as precursors to advanced atherosclerotic plaques [6]. The pathophysiological process involves multiple steps, starting with the inflammatory activation of endothelial cells [6,7,8].

Atherosclerosis and ischemic heart disease in women present unique challenges and considerations compared to men. While atherosclerosis, the buildup of plaque in the arteries, is a common underlying cause of ischemic heart disease in both genders, women often experience different manifestations, risk factors, and outcomes. Unlike men, women tend to develop a non-obstructive atherosclerotic disease in the phase of life preceding menopause due to the action of estrogen on the endothelial response [9].

Myocardial infarction with nonobstructive coronary arteries (MINOCA) is characterized by clinical evidence of acute myocardial infarction (AMI) with normal or near-normal coronary arteries on coronary angiography (stenosis < 50%) and without an alternative diagnosis for the acute presentation. Its prevalence ranges from 6% to 11% among all patients with AMI, with a predominance of young, nonwhite females with fewer traditional risks than those with obstructive coronary artery disease. MINOCA can be due to either epicardial causes such as rupture or fissuring of unstable nonobstructive atherosclerotic plaque, coronary artery spasm, spontaneous coronary dissection and cardioembolism, or other microvascular causes [10].

However, the perception of cardiovascular risk in young women is very poor leading to leading to poor effective prevention action [11,12].

### 2.2. Estrogens Prevent the CVD before Menopause

In the early stages, atherosclerosis may develop without causing significant narrowing or obstruction of the arteries. This stage may be asymptomatic and often goes undetected. However, even non-obstructive atherosclerosis can contribute to inflammation and plaque buildup in the arterial walls [5]. Estrogen, the primary female sex hormone, has both protective and potentially harmful effects on the cardiovascular system [11,12].

Before menopause, estrogen is believed to have a protective effect on the cardiovascular system, as it helps maintain healthy endothelium and vessels by promoting vasodilation, reducing endothelial dysfunction, and reducing vascular aging [9,13]. Estrogen also has antioxidant and anti-inflammatory properties that may reduce the risk of atherosclerosis [13,14]. However, after menopause, estrogen levels decline, leading to changes in lipid metabolism and increased inflammation, and promoting endothelial dysfunction and deposition of visceral fat in the abdomen [9,13,14].

This hormonal change contributes to the progression of atherosclerosis in post-menopausal women and the development of obstructive plaques, leading to a further increase in the risk of cardiovascular events [8,11,12,13].

### 2.3. Estrogen Is Not a Whole Story: The Impact of Lifestyle and (SDOH)

Overall, while estrogen plays a protective role in cardiovascular health before menopause, its decline after menopause may contribute to the progression of atherosclerosis and the development of obstructive ASCVD in women. Knowledge of these hormonal changes and their impact on the cardiovascular system is essential for developing effective prevention and treatment strategies for atherosclerosis in women [1,2]. The progression of atherosclerosis is influenced by both genetic predisposition and exposure to CV risk factors and environmental factors. Over time, atherosclerosis leads to plaque formation and to ASCVD such as ischemic heart disease, Ischemia with Non-Obstructed Coronary Arteries (INOCA), heart attack, stroke, or peripheral artery disease. Adopting a healthy lifestyle, including a balanced diet, regular physical activity, not smoking, and managing chronic conditions such as hypertension and diabetes, can help reduce the risk of developing atherosclerosis and its complications.

## 3. Strategies for CV Primary Prevention in Women

Effectively implementing primary prevention strategies in women poses significant challenges for various reasons. Firstly, there is a widespread belief that cardiovascular disease primarily affects women post-menopause, leading to a skewed perception of risk among young and adult women [11,15]. Secondly, women often face difficulties in adopting healthy lifestyles due to the multitude of social, familial, and occupational obligations they encounter in contrast to men [16,17]. Thirdly, the adoption of healthy habits, particularly physical activity, is heavily influenced by societal, environmental, and structural factors [1,2,3,18]. It is crucial to acknowledge that the development of atherosclerosis begins early in life, emphasizing the necessity for cardiovascular primary prevention to commence at a young age in women [4,5]. Pregnancy presents a unique opportunity to engage young women and guide them toward preventive behaviors [1,2,7,18,19,20].

Unsolved issues in cardiovascular prevention in young women encompass various challenges that persist despite advancements in healthcare. Some of these issues include awareness and perception of cardiovascular risk in young women, gender disparities, socioeconomic factors, lifestyle factors, and psychological and emotional health. Despite the increasing recognition of CVD as a significant health threat to women, there remains a lack of awareness among young women regarding their susceptibility to CVD and the importance of preventive measures [6,21]. Furthermore, women are often underrepresented in cardiovascular research, leading to a lack of understanding of how CVD manifests and progresses differently in women compared to men. This disparity may result in suboptimal prevention and treatment strategies tailored to women’s unique risk factors and health needs [19,22]. Social determinants such as socioeconomic status, education, and access to healthcare significantly influence cardiovascular prevention efforts. Disparities in access to resources and healthcare services may exacerbate risk factors and hinder preventive measures for young women from disadvantaged backgrounds [1,17,19]. Providing access to quality healthcare, including preventive screenings and early intervention for risk factors such as high blood pressure and cholesterol, is crucial in safeguarding cardiovascular health in young women.

Mental health conditions such as stress, anxiety, and depression can impact cardiovascular health and exacerbate risk factors for CVD. Addressing the psychological well-being of young women and implementing effective stress management strategies are essential components of comprehensive cardiovascular prevention efforts [1,15,16,17,18].

Addressing these unsolved issues in cardiovascular prevention in young women requires a multifaceted approach that encompasses education, research, policy changes, and healthcare interventions tailored to the unique needs and challenges faced by this population. Encouraging healthy lifestyle habits such as regular physical activity, a balanced diet, stress management, and avoidance of tobacco and excessive alcohol use can significantly reduce the risk of developing CVD in young women. Additionally, addressing unique factors that affect women’s cardiovascular health, such as pregnancy-related complications like gestational diabetes and pre-eclampsia, is essential.

### 3.1. Pregnancy Stage Is a Good Time to Introduce Primary Prevention in Women

Pregnancy is a challenging moment in the life of a young woman and is characterized by a series of both physical and psychological changes. While changes in physical conditions have been explored in several studies over time, attention to the psychological changes that can affect the body’s responses has only recently increased [2,3,4,5].

The American Heart Association (AHA) proposed a consensus document regarding cardiovascular health in young women before pregnancy [19,20]. The pre-pregnancy period is indeed a critical time for interventions aimed at identifying and managing cardiovascular risk factors in individuals who are planning to conceive. This period of the woman’s life offers an opportunity to address and modify risk factors that may contribute to adverse pregnancy outcomes and subsequent cardiovascular disease for both the mother and the offspring [20,21]. Pregnancy is a very critical moment in a woman’s life and can be burdened by high levels of stress.

As suggested by the AHA consensus, one way to assess lifestyle factors and their impact on cardiovascular health is by evaluating Life’s Simple 7 or Life’s Essential 8 (LS8). Originally defined in 2010, the Life’s Simple 7 framework includes seven key health factors: diet, physical activity, no smoking, body mass index, blood pressure, lipids, and blood sugar. This framework provides a comprehensive approach to assessing and promoting cardiovascular health. In a more recent update, sleep health was included as an eighth factor, resulting in the Life’s Essential 8 framework [20,21,22,23,24].

Furthermore, the consensus document introduces and underlines the importance of the three pillars: stress/resilience, social determinants, and structural policies [20]. These determinants encompass aspects such as mental well-being, socio-economic factors, and access to healthcare and resources, which can influence an individual’s overall health and well-being [20,23,24,25,26]. Stress, anxiety, and depression experienced by mothers during pregnancy harm the development of the fetus and increase the risk of cognitive, behavioral, and emotional difficulties in offspring [27]. At the same time, stress caused by external factors can influence the mother’s lifestyle, especially aspects related to diet and physical activity. Stressful factors during pregnancy can be developing an unplanned or unwanted pregnancy, low economic level, poor support from family and friends, loneliness, and poor medical care support. These stressors can influence the mother’s relationship with food with repercussions on the fetus. To this, we must add the risk of developing pregnancy-related pathologies.

### 3.2. Peripartum Cardiomyopathy

An example of a cardiovascular complication of pregnancy is peripartum cardiomyopathy (PPCM); it is a rare but serious condition characterized by the development of heart failure during the last month of pregnancy or within the first five months postpartum in women without pre-existing heart disease. The exact cause of PPCM is still not fully understood, but it is believed to involve a combination of genetic, hormonal, immunological, and environmental factors.

In the past, PPCM faced challenges in recognition and comprehension, resulting in a deficiency of standardized diagnostic criteria and treatment strategies. Frequently, PPCM was mistaken for other types of heart failure or disregarded due to its rarity and symptom overlap with typical pregnancy manifestations. This lack of awareness and comprehension notably hindered the timely detection and management of affected women, leading to increased rates of morbidity and mortality. The literature on PPCM, characterized by varied and occasionally contradictory discoveries, has posed difficulties for clinicians in providing confident guidance and treatment to patients [28].

The precise cause of PPCM remains elusive, yet it is considered to be multifaceted, involving genetic, environmental, and hormonal elements. This cardiomyopathy is defined by left ventricular systolic dysfunction and heart failure, manifesting in the absence of any other discernible cause. Theories surrounding its origin encompass myocardial inflammation, oxidative stress, and imbalances in angiogenesis. Additionally, PPCM is thought to be associated with the vascular and hormonal alterations inherent in pregnancy [28,29].

Prior research has indicated that many women diagnosed with PPCM experience partial or complete recovery of their left ventricular (LV) function. However, persistent LV systolic dysfunction can lead to adverse cardiac events such as life-threatening ventricular tachyarrhythmias, thromboembolic complications, and even mortality. The rate of recovery from PPCM appears to vary widely among individuals. Unfortunately, there are currently no specific and reliable predictors to determine whether myocardial recovery will occur. One of the factors contributing to the development of PPCM appears to be inflammation [30,31,32,33]. The link between inflammation and stress is very important and could be a good avenue to explore with future research.

## 4. SDOH and Stress in Women

SDOH are conditions in the environments where people are born, live, learn, work, play, worship, and age that affect a wide range of health, functioning, and quality-of-life outcomes and risks [34].

SDOH act by causing an increase in stress. Stress exerts a significant influence on women’s behaviors across various life stages. Societal norms and gender expectations further compound this stress, creating substantial burdens related to caregiving responsibilities, professional duties, and conforming to societal standards of appearance and conduct. These stressors contribute to the risk of developing chronic non-communicable diseases in the future [1,2,21].

Stress can exert profound effects on the cardiovascular system through various physiological mechanisms, including the activation of the sympathetic nervous system, the hypothalamic–pituitary–adrenal (HPA) axis, and inflammatory pathways [25,35,36].

Chronic stress promotes the chronic inflammatory response in the body through the release of pro-inflammatory cytokines and activation of the immune cell system [29,31].

This chronic low-grade inflammation is associated with endothelial dysfunction, oxidative stress, and plaque formation in the arteries, contributing to the development and progression of atherosclerosis. Inflammatory processes in the cardiovascular system increase the risk of coronary and vascular artery disease [11,29,31,37,38].

Stress-induced activation of the sympathetic nervous system and HPA axis can induce endothelial dysfunction.

Endothelial dysfunction is characterized by reduced production of nitric oxide, a potent vasodilator. Impaired endothelial function contributes to vasoconstriction, inflammation, and thrombosis, further exacerbating cardiovascular risk [30,31,32,33,34].

### 4.1. How Stress Influences Diet and Dietary Behavior in Women

The impact of stress on cardiovascular health in women has been rediscovered in the COVID-19 pandemic and post-pandemic period [1,2,25]. Gender variations have been observed in brain activation, autonomic nervous system responses, cortisol secretion, endothelial dysfunction, inflammatory processes, and immune responses. Some studies suggest the amygdala’s role in gender-specific cardiovascular events. Notably, a neuroimaging investigation revealed a correlation between preclinical carotid atherosclerosis and heightened amygdala reactivity [8,39,40].

Stress can have unique effects on women’s health due to various physiological, hormonal, and social factors.

Women’s hormonal fluctuations, particularly during menstruation, pregnancy, and menopause, can influence how they respond to stress. For example, fluctuations in estrogen and progesterone levels can affect mood and stress sensitivity.

Women are more prone to depression and anxiety, which can be exacerbated by stress. Hormonal changes, societal pressures, and caregiving responsibilities may contribute to this increased susceptibility [1]. Societal expectations and gender roles can influence how women experience and cope with stress. Balancing multiple roles such as caregiving, work, and household responsibilities can contribute to chronic stress in women.

Women may use different coping strategies to deal with stress compared to men. Seeking social support, talking about their feelings, and engaging in self-care activities such as exercise, mindfulness, and relaxation techniques are common coping mechanisms for women [1,8,25,41]. Women have a fragility largely determined by the poor socio-economic conditions that lead to incorrect lifestyles and a lack of prevention [1,8,20,25,41].

It is well known that stress can significantly influence dietary patterns and food choices, leading to both short-term and long-term effects on an individual’s eating habits [41]. The impact of stress on diet is complex and can vary from person to person [42]. Many individuals under stress may turn to food for comfort, a phenomenon known as emotional eating. This often involves consuming high-calorie, sugary, or comfort foods as a way to cope with stress and negative emotions [42,43,44,45,46].

Furthermore, stress can induce food craving [47]. Food craving is characterized by a strong urge to consume a particular food. In Western cultures, these foods typically boast high palatability and are energy-dense, often containing high levels of sugar and/or fat. This craving experience encompasses various dimensions, including cognitive (such as thinking about food), emotional (like the desire to eat or mood changes), behavioral (such as seeking and consuming food), and physiological (including salivation) aspects [47].

These cravings may be linked to the brain’s response to stress hormones [44,46,47,48,49]. Stress triggers the release of hormones, such as cortisol, which can impact metabolism and lead to weight gain, especially around the abdominal area. This hormonal response can influence the body’s storage of fat [42,45].

Stress can lead to disruptions in normal eating patterns [41,42,43,44,45,46]. Some people may experience an increase in appetite and overeat, while others may lose their appetite and undereat. Both patterns can have consequences for overall health, especially during pregnancy.

### 4.2. Stress during Pregnancy and Its Relationship with Food

Several studies that have analyzed the impact of stress during pregnancy highlight the fundamental role of nurses who care for mothers in suggesting adequate nutrition and methods to reduce anxiety and stress [50,51].

Celik and coworkers investigated the stress, emotional eating, and weight bias levels in 210 Turkish pregnant women [50]. They found that pregnant women experience a moderate level of stress, emotional eating, and weight bias. There was a significant relationship between the weight bias score averages and the emotional eating and stress score averages of the pregnant women. The study underlines that nearly 1 in 2 pregnant women was overweight or obese, and when the body mass index level of the women increased, their weight stigma and emotional eating also increased. The authors underlined the importance of the nurse in providing training and counseling on how to deal with stress, stigma, and nutrition during pregnancy, in order to ensure the psychological adaptation of pregnant women to childbirth and the postpartum period, which they are at risk of in terms of stress and emotional problems. Bias factors are related to nutrition and weight [50].

Jackson H and coworkers evaluated pregnant women’s recall of the nutritional advice provided by their healthcare during pregnancy [52]. They found that approximately half of the women enrolled in the survey recall receiving nutritional counseling during pregnancy and that 73% of women who were counseled on nutrition changed their behavior based on the recommendations received [52].

Pregnancy and nutrition are closely intertwined, as proper nutrition is essential for the health and well-being of both the mother and the developing baby. The first crucial point is a balanced and varied diet that includes a wide range of nutrients [45,53,54,55,56]. The diet should include fruits, vegetables, whole grains, lean proteins, dairy or plant-based alternatives, and healthy fats. It is important to avoid foods with empty calories and focus on nutrient-rich options [45,53,54,55,56]. Little information is available on the effect of stimulants such as coffee and caffeine during pregnancy. The guidelines indicate that a dosage of caffeine up to 200 mg per day is to be considered safe in women who are habitual consumers [57,58,59,60]. However, some studies link high caffeine intake with an increase in anxiety [61,62,63,64].

Many of the potential benefits attributed to coffee stem from the belief that it possesses antioxidant and anti-inflammatory properties [65]. The primary constituents of coffee believed to exert such effects include phenolic compounds, caffeine, diterpenes, trigonelline, and melanoidins [57,65]. Among these, the phenolic component is predominantly characterized by chlorogenic acids. Chlorogenic acids have been shown to elicit antioxidant effects by reducing the production of inflammatory mediators through various mechanisms [66]. The anti-inflammatory properties of coffee might also be influenced by its impact on the gut microbiota. Pre-clinical and human studies suggest that consuming coffee can lead to alterations in the composition and activity of the gut microbiota. This can result in changes to the balance among major microbial phyla favoring a profile associated with anti-obesity effects [67].

Social expectations require women to adopt a healthy lifestyle during pregnancy; however, no univocal behavior emerges in the various studies. The reduction in the consumption of coffee and tea appears frequent even if not supported by data highlighting side effects caused by moderate consumption of these drinks [68]. There are no studies that evaluate the consumption of energy drinks in young pregnant women; this is an interesting point to develop in the future considering the significant increase in the consumption of these caffeinated drinks [69,70].

A very controversial point is the impact of a vegan or vegetarian diet on cardiovascular health and prevention. These diets have been little studied compared to the Mediterranean diet [71]. The quality of the plant-based diet is an important feature. Individuals who follow a nutritious plant-based diet reportedly exhibit lower body mass index, reduced waist circumference, and decreased visceral fat compared to those adhering to less healthful plant-based diets [72]. Researchers have observed that the quality of the diet might hold greater significance than specific dietary patterns when comparing vegans, vegetarians, and omnivores, as there are no significant differences in adiposity values among these groups [73]. A recent meta-analysis on the vegetarian diet found that consuming a vegetarian diet was associated with significant improvements in low-density lipoprotein cholesterol (LDL-C), glycated hemoglobin (HbA1c), and body weight beyond the standard therapy in individuals at high risk of CVDs. However, the changes in LDL-C and HbA1c did not reach the clinical significance as per the cutoff target [74]. Few studies have been conducted on the effects of a vegetarian diet on cardiovascular risk in primary prevention. During pregnancy, blood docosahexaenoic acids (DHA) concentrations are often lower in vegetarians than in nonvegetarians; cord blood DHA is lower in infants of vegetarians [75]. When food access is satisfactory, infant birth weights and the duration of gestation are similar in vegetarian and nonvegetarian pregnancies [62], leading to the conclusion that with adequate nutrient intake, vegetarian and vegan diets are safe in pregnancy.

Stress can contribute to mindless or distracted eating, where individuals may eat without paying attention to hunger cues or the nutritional content of the food [75,76].

### 4.3. Perinatal Depression and Its Relationship with Food

Perinatal depression is defined as the onset of a major or minor depressive episode during pregnancy (antenatal depression), after childbirth (postpartum depression), or both [76].

Christian and coworkers explored the variations in eating disorders or depression symptoms among women at different stages of pregnancy and the impact of social determinants [77]. During pregnancy, eating disorder symptoms and various social and self-evaluative factors were uniquely linked to depression. Specifically, eating disorder symptoms, maladaptive perfectionism, social appearance anxiety, and self-compassion during pregnancy were significant predictors of postpartum depression, even after considering prenatal depression. Notably, during pregnancy, but not postpartum, a stronger association between eating disorders and depression symptoms was observed when social support and self-compassion were low and maladaptive perfectionism was high [77]. The authors suggest that targeting eating disorder symptoms and addressing social and self-evaluative factors in routine medical care and stepped-care interventions could enhance maternal mental healthcare and prevent postpartum depression [77]. The transition from pregnancy to postpartum represents a critical phase marked by numerous significant and abrupt changes. These include weight gain, alterations in appetite, fluctuations in hormones, and changes in social relationships. These biopsychosocial transformations, combined with increased social pressures and expectations surrounding pregnancy, such as the desire to appear “glowing” and the pressure to swiftly return to pre-pregnancy weight, contribute to the risk of experiencing alterations in eating behavior and depressive symptoms.

Support from healthcare professionals plays a fundamental role in helping women in the postpartum phase, reducing performance anxiety and expectations, and reducing the risk of developing depression. Moreover, a significant association between antenatal depression and the development of new CVD within 24 months postpartum exists [78,79,80,81].

Recent research has indeed highlighted the significant role of dietary and nutritional interventions in reducing depression, partly through their effects on the gut microbiota [82,83]. A diet rich in fruits, vegetables, whole grains, lean proteins, and healthy fats is associated with a lower risk of depression. These foods provide essential nutrients and antioxidants that support brain health and reduce inflammation, which is implicated in depression [41,84]. Conversely, diets high in processed foods, sugars, and unhealthy fats can contribute to inflammation and increase the risk of depression [41,84]. The gut microbiota, composed of trillions of microorganisms living in the digestive tract, plays a crucial role in brain health and mood regulation. Dietary patterns can influence the composition and diversity of the gut microbiota. A diet high in fiber and fermented foods, such as yogurt, kefir, sauerkraut, and kimchi, promotes the growth of beneficial gut bacteria, which can positively impact mood and reduce symptoms of depression [41,84]. Prebiotics are nondigestible fibers that serve as food for beneficial gut bacteria. Studies suggest that probiotic supplements and foods containing probiotics, such as yogurt and kefir, may have antidepressant effects by modulating the gut–brain axis and reducing inflammation [85]. Several studies have shown that omega-3 supplementation may reduce symptoms of depression and improve mood regulation. Omega-3 may also influence the gut microbiota composition, contributing to their antidepressant effects [86].

Some micronutrients, including vitamins B6, B12, folate, vitamin D, and magnesium, play key roles in neurotransmitter synthesis, mood regulation, and stress response. Deficiencies in these nutrients have been linked to an increased risk of depression.

The prevalence of vitamin D deficiency among pregnant women and newborns is a cause for serious concern as vitamin D plays a crucial role in hippocampal learning and memory for mothers and in neural cell growth for offspring as shown in preclinical studies [87,88]. Vitamin D acts as a neuroactive hormone, influencing the concentration of neuronal calcium ions, which are key to regulating neuroplasticity and mood [89,90].

A meta-analysis of nine longitudinal studies involving 8470 subjects revealed a noteworthy inverse association between serum 25(OH)D levels and the risk of postpartum depression, with a cutoff of 50 nmol/L [91]. However, a randomized controlled trial indicated that supplementation with 2000 IU vitamin D3 from 26 to 28 weeks of gestation up to childbirth significantly reduced depression scores [92]. These findings suggested that vitamin D supplementation during late pregnancy could be advantageous in mitigating perinatal depression [92,93].

Overall, dietary and nutritional interventions can have potent effects on reducing depression, partly through their interactions with the gut microbiota. Prolonged or chronic stress may affect the absorption of nutrients in the digestive system, potentially leading to nutrient deficiencies over time and inducing changes in the microbiota. The microbiota is a new cardiovascular risk factor that influences the absorption of fats and some drugs such as oral hypoglycemics and statins [94,95,96].

All this evidence supports the hypothesis that counseling in women plays an important role in maintaining and undertaking correct lifestyles [2,7,97]. The central component of the lifestyle is the diet, which is able to influence the other aspects of a healthy life such as obesity and sleep quality. The Academy of Nutrition and Dietetics identifies a balanced diet and adequate weight gain as two important components of a healthy pregnancy [98]. The amount of food a woman needs during pregnancy depends on a number of things including her body mass index, before pregnancy, the rate at which she gains weight, age, and appetite. All pregnant women should eat a variety of nutrient-rich foods each day [52,53,54,56,98].

These psychological conditions can have negative effects on the health of the mother during pregnancy and increase the risk of complications. Furthermore, they have a long-term effect that is reflected in the development of non-communicable diseases in adult women [24,99,100].

## 5. Proposed Intervention for Prevention in Young Women: The WEAR-Being Project

The WEAR-being project was designed to collect information on cardiovascular health in women in different age groups. For this end-point, young women and pre-menopausal women were selected. The goal is to gain information about cardiovascular health and how it is perceived in young women and women approaching menopause. The second objective of the study is to implement a lifestyle correction model that is personalized, responds to the needs of the individual woman, and is pursued through direct coaching carried out by expert and dedicated healthcare personnel. The third objective is to collect objective data through precise tools on the cardiovascular responses during the lifestyle modifications proposed by the trainers and verify whether this approach is effective.

The WEAR-being project addresses the global challenge of maintaining health in a rapidly changing society (HEALTH Cluster—Horizon Europe strategic plan) by providing lifestyle monitoring in a group of young women (30–40 years) compared with a control group of menopausal women (50–60 years). The devices will record data relating to daily physical activity, quality and duration of sleep, and vital signs. Information on calorie intake and diet quality will be entered manually by the subjects. The data collected will be managed through Big Data management techniques and will be analyzed using artificial intelligence and data mining techniques in order to establish personal trends and ranges of values in relation to the diet and activity carried out by the subject.

For this purpose, a 12-month monitoring study has been developed which will involve women. The women will be recruited through campaigns conducted on social media and through charity foundations that are very active in the area. The analysis will evaluate adherence to a correct lifestyle (diet, physical activity, and stress levels) and the control of bio-humoral parameters and vital parameters. For the objective assessment of lifestyle, the Life’s Essential 8 evaluation score will be used. Women will be sent to dietary and lifestyle counseling sessions. The counseling sessions will be carried out in small groups and personalized and guided by specifically trained healthcare professionals. In young women, the need for good cardiovascular health will be highlighted in order to preserve the health of the fetus in the event of a pregnancy. Counseling dedicated to stress management will be introduced. The project is under evaluation by the local Ethics Committee. The study was developed in accordance with the Helsinki Declaration (www.wma.net (accessed on 1 December 2023)), and all patients will provide informed consent. The data will be processed in accordance with current privacy legislation. The goal is to promote awareness of good cardiovascular health even in young women. We have identified the Mediterranean Diet which is easily achievable in our country. The Mediterranean score and Life’s Essential 8 score will also be calculated and compared in the same subject in order to verify an improvement in behavior and take personalized actions. To encourage subjects to increase physical activity, a wearable device monitoring activity (i.e., number of kilometers walking/day) will be used [101]. Over the past few years, wearable technologies have become increasingly common in everyday life. From an industry study conducted by Gartner, it has been found that the device market of wearables is continuously growing with an estimated spending of USD 81.5 billion in 2021 [102]. Wearable devices are a high-tech solution that can help us in the promotion of well-being, playing an important role in monitoring physical activity, diet and rhythms sleep, anxiety, and stress [103,104]. Furthermore, they allow the person wearing them to interact and monitor their body throughout the day, increasing awareness and active involvement in improving one’s well-being. In a research study conducted by Business Insider, 75% of users agreed that wearable devices promote encouragement to take care of one’s health, thereby promoting virtuous behavior [103,104]. Healthcare providers play a critical role in educating and supporting pregnant women in making healthy choices, monitoring weight gain during pregnancy, and managing any underlying health conditions. We aim to increase this attention towards young women. The WEAR-being project will contribute to stimulating awareness of the relevance of cardiovascular primary prevention in young women.

## 6. Conclusions

In conclusion, the social determinants of health have a strong impact on lifestyle, and women are more likely to adopt unhealthy behaviors in response to stress. Diet is an excellent tool to guide the trend toward healthy behaviors. To prevent chronic non-communicable disease, it is advisable to start primary prevention through correct lifestyles at a young age, and among women, a good time to intercept is pregnancy. Pregnancy can be a challenging factor in a woman’s life because she is more exposed to stressful situations. Healthcare personnel, mainly nurses, play an important educational role in educating and supporting young women, especially from disadvantaged social backgrounds.
